# The faecal microbiome and carriage of *Salmonella* in Australian captive pythons

**DOI:** 10.1093/femsec/fiag084

**Published:** 2026-07-29

**Authors:** Amreeta Sarjit, Aaron Mc Hall, Naveen Sellapperumage, Chloe Makings, Gary A Dykes

**Affiliations:** Institute of Innovation, Science and Sustainability, Federation University, Churchill, Victoria 3842, Australia; Institute of Innovation, Science and Sustainability, Federation University, Churchill, Victoria 3842, Australia; Institute of Innovation, Science and Sustainability, Federation University, Churchill, Victoria 3842, Australia; Institute of Innovation, Science and Sustainability, Federation University, Churchill, Victoria 3842, Australia; School of Agriculture and Food Sustainability, University of Queensland, St Lucia, Queensland 4072, Australia

**Keywords:** Australia, biofilms, microbiome, polystyrene, pythons, *Salmonella*

## Abstract

There is a growing interest in keeping exotic pets such as pythons in Australia. Reptiles are known asymptomatic carriers of *Salmonella* in their digestive tract. This study investigated the microbiome and carriage of *Salmonella* in faeces from captive Australian pythons (*n* = 28). This study also aimed to determine the effect of temperature and nutrient availability on monospecies biofilm formation of python-associated *Salmonella* isolated in this study (*n* = 10) as a possible indicator of persistence. A total of 23 *Salmonella* strains were identified from 28 captive Australian pythons, representing diverse serovars of subspecies *enterica*, namely *S*. Bergedorf, *S*. Havana, *S*. Jangwani, *S*. Java, *S*. Kottbus, *S*. Muenchen, and *S*. Umbadah, as well as subspecies *S. diarizonae* and *S. salamae*. The composition of the microbial communities differed according to sampling location, diet, and *Salmonella* serovars or subspecies. Bacillota, Bacteroidota, and Pseudomonadota were the most abundant taxa across the faecal microbiome. Strains belonging to subspecies *enterica* were generally poorer biofilm formers than strains belonging to subspecies *salamae* and *diarizonae*. This study shows that microbial communities are variable depending on the environmental factors, which could increase the risk of acquiring reptile-associated salmonellosis while handling captive pythons.

## Teaser

This study is the first integrated study of the faecal microbiome and genomic characterization of Salmonella in Australian captive pythons.

## Introduction

Reptiles such as pythons are common asymptomatic carriers of *Salmonella*. Human *Salmonella* infections from captive reptiles known as reptile-associated salmonellosis (RAS) are of public health concern. Transmission occurs through direct or indirect contact with infected animals, their excreta, or contaminated environments (Pees et al. 2023). While many animals serve as reservoirs, reptiles are of particular concern due to their capacity to asymptomatically harbour *Salmonella* and shed it into surrounding environments. Such infections occur through handling of reptiles and are specifically prevalent in children with reptiles as pets. There is an increasing interest in keeping captive-bred native reptile species as pets in Australia. Children tend to keep pet lizards and turtles (Marin et al. 2021, Pees et al. 2023). Adults are known to have a growing interest in keeping captive snakes as compared to children due to the intensity of handling snakes and environmental management required (Kusrini et al. 2021). Handling reptiles enhances the risk of RAS (Pees et al. 2023). A survey reported that 3% of Australian households have ∼ 538 000 reptiles as pets, with the most popular pet reptiles being snakes (36% of the pet reptiles) followed by tortoises (33% of the pet reptiles) and then lizards (31% of the pet reptiles) (Animal Medicines Australia 2022). While wild reptiles may naturally harbour *Salmonella* as part of their intestinal microbiota, captivity-related factors such as stress, diet, and enclosure hygiene can exacerbate bacterial shedding, thereby increasing the potential for zoonotic transmission (Corrente et al. 2017, Pees et al. 2023).

The *Pythonidae* family, which consists of 11 genera is commonly reported in Africa, South-East Asia and Oceania including Australia (Kyle and Downs 2025). Native captive-bred pythons are allowed as pets in Australia. The reptilian gut microbiome is a complex and dynamic ecosystem shaped by host physiology and environmental factors. In captive reptiles, dietary differences such as rodent or bird-based diets may impact the relative abundance of dominant phyla, particularly Bacillota, Bacteroidota, and Pseudomonadota, which play key roles in nutrient metabolism and immune regulation (Xiang et al. 2025). As ectotherms, reptiles are also highly sensitive to temperature fluctuations, with even minor environmental changes capable of inducing instability or shifts in microbial structure, making the gut microbiome a potential indicator of health and husbandry quality. Reptiles maintain body temperatures around 25 °C, as demonstrated by Brattstrom (1965), who reported a mean body temperature of 25.6 °C across 161 reptile species. Reptile enclosures consist of a basking spot temperature of ∼35 °C–37 °C. These temperatures may support *Salmonella* survival and biofilm development on external surfaces in reptile enclosures such as reptile skin and polystyrene. Biofilms form communities of monospecies or multispecies microbial cells encased in an extrapolymeric matrix on a specific surface (Costerton et al. 1999).

To our knowledge, there is very limited work conducted on *Salmonella* in captive pythons in Australia and worldwide (Muslin et al. 2025). Little work has also been conducted on biofilms of *Salmonella* derived from captive pythons. This study was conducted to (i) isolate *Salmonella* from faeces of captive pythons (*n* = 28) followed by the characterization of *Salmonella* using whole genome sequencing, (ii) determine the microbiome of the matching faecal samples from the same pythons, (iii) analyse the relationship between the diet of captive pythons, microbiome composition and variation in *Salmonella* serotypes and subspecies using *in silico* tools and to (iv) investigate monospecies biofilm formation of *Salmonella* at 25 °C and 37 °C on polystyrene. By integrating whole-genome sequencing with microbiome profiling, this work highlights how host-associated and environmental factors influence *Salmonella* persistence and transmission. To our knowledge, this is the first study that investigated the faecal microbiome of Australian captive pythons and its influence on the carriage and biofilm formation of *Salmonella*.

## Materials and methods

### Sample collection

Faecal samples of captive pythons were collected from pet owners across Victoria and Queensland (*n* =28). Pet owners were recruited for this study through personal networks, herpetology societies, and online reptile owner communities. Owners were instructed on aseptic techniques of collecting samples. Sampling location refers to the location of owner residence. A single faecal sample was collected from each python. Faecal samples were either dry or wet as it was collected within 24 h of excretion. The type of python and its predominant diet were recorded. Samples were kept cold on icepacks while transferred after collection for processing at the laboratory. Faecal samples were then processed at the laboratory as described previously by Pao et al. (2005). Briefly 1 g of faeces was pre-enriched in 9 ml of Buffered Peptone Water (BPW; Oxoid, UK) at 37 °C for 24 h. Then, 0.1 ml of the culture was inoculated onto the selective enrichment Rappaport-Vassiliadis Broth (RV; Oxoid, UK) at 42 °C for 24 h. The enriched cultures were then inoculated onto Xylose Lysine Deoxycholate (XLD; Oxoid, UK) agar using the streak plate method where it was incubated at 37 °C for 24 h. Presumptive *Salmonella* isolates on XLD exhibited black centred colonies.

### Whole genome sequencing of *Salmonella*

*Salmonella* genomic DNA (*n* = 23) was extracted using Wizard® Genomic DNA Purification Kit (Promega, USA) in accordance with manufacturer’s directions. The genomic DNA was fragmented and tagged using a Nextera XT DNA library preparation version 3600-cycle MiSeq kits followed by whole-genome sequencing on an Illumina Miseq i100 plus Sequencer (300 bp paired-end reads) as described previously by Sarjit et al. (2021). Briefly, genomes were assembled using SPAdes v4.0.0 and annotated using Prokka v1.14.6 or RAST toolkit (RASTk). All genomes have been deposited in GenBank in the National Centre for Biotechnology Information (NCBI) website accessible at https://www.ncbi.nlm.nih.gov/ with the following BioProject IDs, PRJNA1432403, PRJNA1433053, PRJNA1433056, PRJNA1433063, and PRJNA1433138. *Salmonella* strains were serotyped using SeqSero2 (Zhang et al. 2019). Pan-genome analysis was conducted using the Roary tool v3.13.0 with an identity cutoff of 95% to determine the presence and absence of genes across the *Salmonella* strains (Page et al. 2015). Variation within the gene content was also determined in RASTk to determine the differences in gene function across these strains. The genome feature files (GFF3) generated from Prokka were applied to determine the pan-genome of the strains investigated in this study. Screening of 14 genes related to stress response towards environmental factors was investigated from the GFF3 files produced from the pan-genome as described previously (Nickerson and Curtiss 1997, Miticka et al. 2003, Chen et al. 2014, Mayola et al. 2014, Page et al. 2015, Ricke et al. 2018, Ray et al. 2019, Li et al. 2024). The presence /absence of these selected 14 genes was further confirmed using BLASTn with a maximum e value of 10^−30^ (Altschul et al. 1997, Sarjit et al. 2023). The functions of these genes are described in Table S1 in the Supplementary Information. Comparative genomic analyses were performed by calculating pairwise Average Nucleotide Identity (ANI) values using FastANI v1.3, while hierarchical clustering and phylogenetic trees were constructed from distance matrices to visualise genomic relatedness (Jain et al. 2018). ANI values were determined using FastANI to determine the average values of similarities between common homologous regions within genomes (Seribelli et al. 2020). It also determines the genome-level similarity between the strains investigated in this study. A Principal Component Analysis (PCA) based on single-nucleotide polymorphism (SNP) alignments was generated by Snippy v4.6.0 to assess genetic variation and clustering among strains according to serovar and sampling location (Seemann 2015). To determine potential transmission of *Salmonella*, inStrain v 1.10.0 was used to compare the phylogeny of *Salmonella* based on SNP and ANI (Olm et al. 2021). InStrain compare v1.5.3 was used to produce an ANI dendogram based on the pairwise population ANI distance matrix. In addition, inStrain compare also produced a consensus SNP tree based on the pairwise consensus SNP differences among strains.

### 16S rRNA Sequencing and bioinformatics analysis

DNA of matching faecal samples was extracted using QIAamp PowerFecal DNA Kit (Qiagen, Valencia, CA, USA) in accordance with the manufacturer’s instructions. The preparation of a 16S rRNA Library targeting the V4 region, followed by sequencing of amplicons was conducted through the Illumina MiSeq i100 plus platform (Illumina, San Diego, CA, USA) with a paired-end 300 base pair sequencing protocol as described previously (Kang et al. 2019). Paired end reads were clustered to form operational taxonomic units with a cut off at 97% from identification through the use of the MOTHUR pipeline, taxonomic classification was processed in SILVA 16S rRNA database, PRIMER-7 was used to identify bacterial arrangements, multivariate roles and any non-metric multidimensional scaling as described previously (Schloss et al. 2009, Quast et al. 2012, Clarke and Gorley 2015, Kang et al. 2019). Although there has been a drift in analysing reads as amplicon sequence variants (ASVs) rather than clustering reads into OTUs, this study still adopted the use of OTUs as the clustering of reads into OTU’s decreases sequencing errors. A similar output is achieved using the denoising method for ASVs using DADA2 and clustering method for OTUs using MOTHUR (Chiarello et al. 2022, Segura et al. 2024). Previous studies have also reported comparable alpha and beta diversity results using both ASVs and OTUs (García-López et al. 2021). Microbial diversity analysis was conducted in RStudio v2025.09.1+401 using the Phyloseq v1.52.0, Vegan v2.7.1, and Tidyverse v2.0.0 packages, with Alpha diversity assessed via Shannon, Simpson, Chao1, and observed richness indices, and Beta diversity estimated using the Bray–Curtis dissimilarity index. Heatmaps, dendrograms, and ordination plots were produced using ggplot2 v4.0.0, pheatmap v1.0.13, scales v1.4.0 and ComplexHeatmap v2.26.1 to visualize clustering patterns by sampling location, diet, and *Salmonella* serovar. A Principle Coordinates Analysis (PCoA) plot to determine the compositional biplot was determined using the R package (Palarea-Albaladejo and Martín-Fernández 2015). The raw sequences of the microbiome data have been deposited in NCBI with the following BioProject ID PRJNA1432430.

### Bacterial strains and growth conditions for biofilm studies

Ten strains of *Salmonella* characterized and identified using whole-genome sequencing and *in silico* tools from the section above were selected for biofilm studies. These strains were selected based on their serovar, source, and location of isolation. These included subspecies *enterica* (*n* = 7), subspecies *diarizonae* (*n* = 2), and subspecies *salamae* (*n* =1). Amongst the subspecies *enterica*, there were *S*. Jangwani S1A (*n* = 1), *S*. Java S2A (*n* = 1) *S*. Muenchen S9A and S12A (*n* = 2), *S*. Havana S7A and S14A (*n* = 2), and *S*. Umbadah (*n* = 1). Both *S*. Typhimurium ATCC 14028 and ATCC 13311, which are non-reptilian strains, were used as reference strains in this study. Bacterial cultures were grown aerobically at 37 °C for 24 h on xylose lysine deoxycholate (XLD) agar (Oxoid, UK) or in tryptic soy broth (TSB; Oxoid, UK) unless otherwise stated. Each *Salmonella* strain was cultured in TSB at 37 °C for 24 h. *Salmonella* suspensions were diluted in either full strength or half strength TSB to obtain a cell density of ∼10^4^ CFU ml^−1^, as described previously (Sarjit and Dykes 2015).

### Biofilm formation using culture-based techniques and crystal violet assay

Polystyrene coupons (76 mm × 24 mm × 0.4 mm) were placed in 20 ml of bacterial suspensions of each *Salmonella* strain at approximately 10^4^ CFU ml^−1^ as described in the section above. Coupons were incubated at 25 °C and 37 °C, respectively, for 24 h to allow biofilm formation. The biofilm formation assay using the culture-based technique was conducted as Sarjit and Dykes 2017. Coupons were cleaned as described previously (Wang et al. 2024). For the microtitre plate assay, it was conducted according to Chen et al. (2021).

Plates were incubated at two temperature conditions, 25 °C and 37 °C for 24 h for biofilm formation. The absorbance readings of the plates were obtained using a microplate reader at 595 nm. Specific Biofilm Formation (SBF) values were calculated to quantify surface-adhered biofilm biomass relative to overall bacterial growth as described previously (Teh et al. 2019)


\begin{eqnarray*}
SBF = \frac{{OD\,\,of\,\,\mathit{crystal}\,\,\mathit{violet}\,\,\mathit{stained}\,\,\mathit{wells} - OD\,\,of\,\,\mathit{control}\,\,\mathit{wells}}}{{OD\,\,of\,\,\mathit{bacterial}\,\,\mathit{growth}\,\,\mathit{prior}\,\,to\,\,\mathit{staining}}}.
\end{eqnarray*}

### Statistical analysis

Statistical significance among diversity measures was tested using Kruskal–Wallis and Wilcoxon rank-sum tests for alpha diversity and permutational ANOVA (PERMANOVA) for beta diversity, with *P* ≤ 0.05 considered significant. Since PERMANOVA is sensitive to both centroid groups and heterogeneity of dispersion, a permutational analysis of multivariate dispersion (PERMIDISP) was conducted as described previously (Hole et al. 2023). Kruskal–Wallis and Wilcoxon rank-sum tests were used to assess differences in alpha diversity indices (Shannon, Simpson, observed richness, and Chao1) among groups. Microbiome Multivariable Association with Linear Models2 (MaAsLin2) using general linear and mixed models was conducted to determine the differences in relative abundance of the *Enterobacteriaceae* family with *Salmonella-positive* samples (Mallick et al. 2021). All analyses were performed in R v4.3.1, and workflows were managed through version-controlled Galaxy available at https://doi.org/10.1093/nar/gkae410 and GitHub repositories available at https://github.com/tseemann/snippy , https://github.com/ekg/vcflib, https://github.com/shenwei356/csvtk and https://github.com/smdabdoub/kraken-biom to ensure reproducibility and data integrity.

All the biofilm assays were carried out in triplicate with independently grown *Salmonella* cultures and the values were expressed as the mean ± standard deviation. A two-way analysis of variance (ANOVA) using a post hoc Tukey’s test was conducted to assess the effect of temperature and nutrient availability on biofilm formation. The statistical analysis was performed using SPSS software (SPSS 29 software (IBM Inc., Armonk, NY, USA) using a 95% confidence level.

## Results

### Isolation and whole-genome sequencing of *Salmonella*

**Figure 1:**
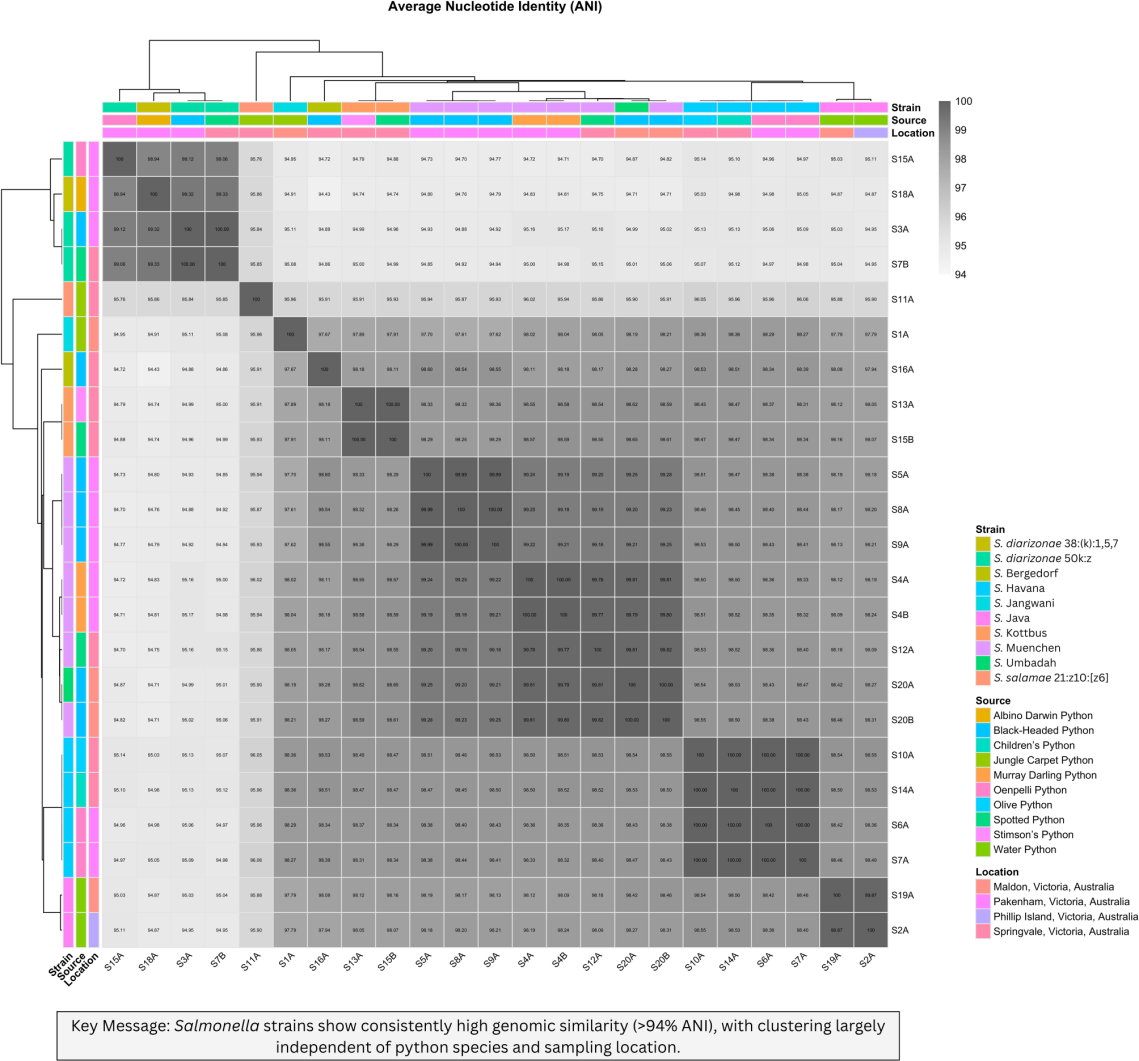
ANI among *Salmonella*. Hierarchical clustering was performed using pairwise ANI values. Annotation bars indicate the *Salmonella* serovar (strain), python species (source), and sampling location. Numbers in heatmap represent percentage of genomic similarity.

Out of 28 samples, *Salmonella* was detected in 19 out of 28 (68%) faecal samples collected in this study. Across the different types of pythons, *Salmonella* was only not isolated from the *Aspidites ramsayi* (woma python). The source, diet, date of isolation, location of the captive pythons, strain ID, and owner IDs are shown in Table S2. There were 23 strains of *S. enterica* characterized in this study. At the subspecies level, 18 of the strains were subspecies *enterica* with serovars *S*. Bergedorf, *S*. Havana, *S*. Jangwani, *S*. Java, *S*. Kottbus, *S*. Muenchen, and *S*. Umbadah. The other subspecies isolated were subspecies *diarizonae* and *salamae*. Among the strains that were characterized, *S*. Muenchen was the most dominant serovar detected, followed by *S*. Havana, *S*. Java, and *S*. Bergedorf detected across captive python samples. Among all the samples, two strains each were successfully isolated from four different pythons. This included *S*. Muenchen (S4A and S4B) from *Morelia spilota* (Murray Darling python), *S*. Havana S7A, and *S. diarizonae* 50k: z S7B from *Morelia oenpelliensis* (Oenpelli python)*, S. diarizonae* 50k: z S15A, and *S*. Kottbus S15B from *Antaresia maculosa* (spotted Python) and *S*. Umbadah S20A and *S*. Muenchen S20B from *Aspidites melanocephalus* (black-headed Python). Figure S1 shows the principal component analysis (PCA) of *Salmonella* strains to assess genetic variation based on SNP among different serotypes and subspecies. Gene annotations using SNP identified differences within a range of protein-coding genes such as *gyrB, dsdA, rbsK, fucP, dagR*, and *mgtA*. These genes are associated with DNA replication, metabolism carbohydrate utilization, transcriptional regulation, and ion transport, functions that may contribute to environmental adaptation, persistence, and survival within captive python environments. The first two principal components (PC1 = 58.05% and PC2 = 13.53%) together explain 71.58% of the total genomic variance. This analysis highlights clear genomic differentiation among several *Salmonella* serotypes, with *S*. Havana, *S*. Java, and *S*. Muenchen forming distinct clusters compared with other serovars. The separation along PC1 primarily distinguishes the *S. diarizonae* and *S*. Umbadah strains from the main cluster, suggesting unique genomic features or evolutionary divergence within these lineages. The heatmap of the average nucleotide identity (ANI) across all *Salmonella* strains based on sampling location and type of python is shown in Fig. 1. Figure 1 shows that *Salmonella* strains were highly similar, with pairwise ANI values ranging from ∼94% to 100%. *S*. diarizonae (S15A, S18A, S3A, and S7B) clustered closely together based on the inStrain SNP and ANI phylogenetic tree shown in Fig. S2, exhibiting near-identical genomic profiles, suggesting high degree of genetic relationship between these strains. All these strains were of the same subspecies but different serovars, isolated from different types of pythons, diet and sampling location. The largest cluster comprised S5A, S8A, S9A, S4A, S4B, S12A, S20A, and S20B in Fig. 1, all of which exhibited ANI values exceeding 99%, indicating very close genomic relatedness. Clustering appeared to be more consistent with *Salmonella* serovars, suggesting that serovars exert a stronger influence on genomic relatedness compared to either host species or geographic sampling location for captive python populations.

The pan-genome analysis of all *Salmonella* strains revealed substantial genomic diversity, comprising a total of 15 106 genes. Of these, 3057 genes (20.2%) were identified as core genes, consistently present across all strains, representing fundamental cellular functions. In contrast, 7651 genes (50.6%) were classified as accessory genes, found in two or more but not all strains, highlighting the genomic flexibility and potential for adaptation to different hosts or environments. Additionally, 4398 genes (29.1%) were unique, being present in only a single strain, which highlights the presence of strain-specific genetic elements that may contribute to specialization or distinct virulence profiles. Key virulence genes involved in stress response (*grpE, dnaK, rpoH, rpoS*), osmoregulation (*proV, otsA, otsB*), cell adhesion, and outer membrane integrity (*cfa, ompC, ompF*), lipid metabolism (*fabA*), and DNA repair or global regulation (*recA, rpoE, cspA*) were reported across all genomes in this study. There were higher number of accessory genes compared to core genes in this study. This could be due genomes being more open due to selective pressure. However, genes associated with stress response located in the core genome was selected as a choice of focus in this study due to its association with cellular function in *Salmonella*. In addition, the presence of these stress response genes may enhance their survival ability on faecal samples of pythons under varying environmental conditions through adaptations in the surrounding environment.

### Microbial composition of captive python faeces

**Figure 2:**
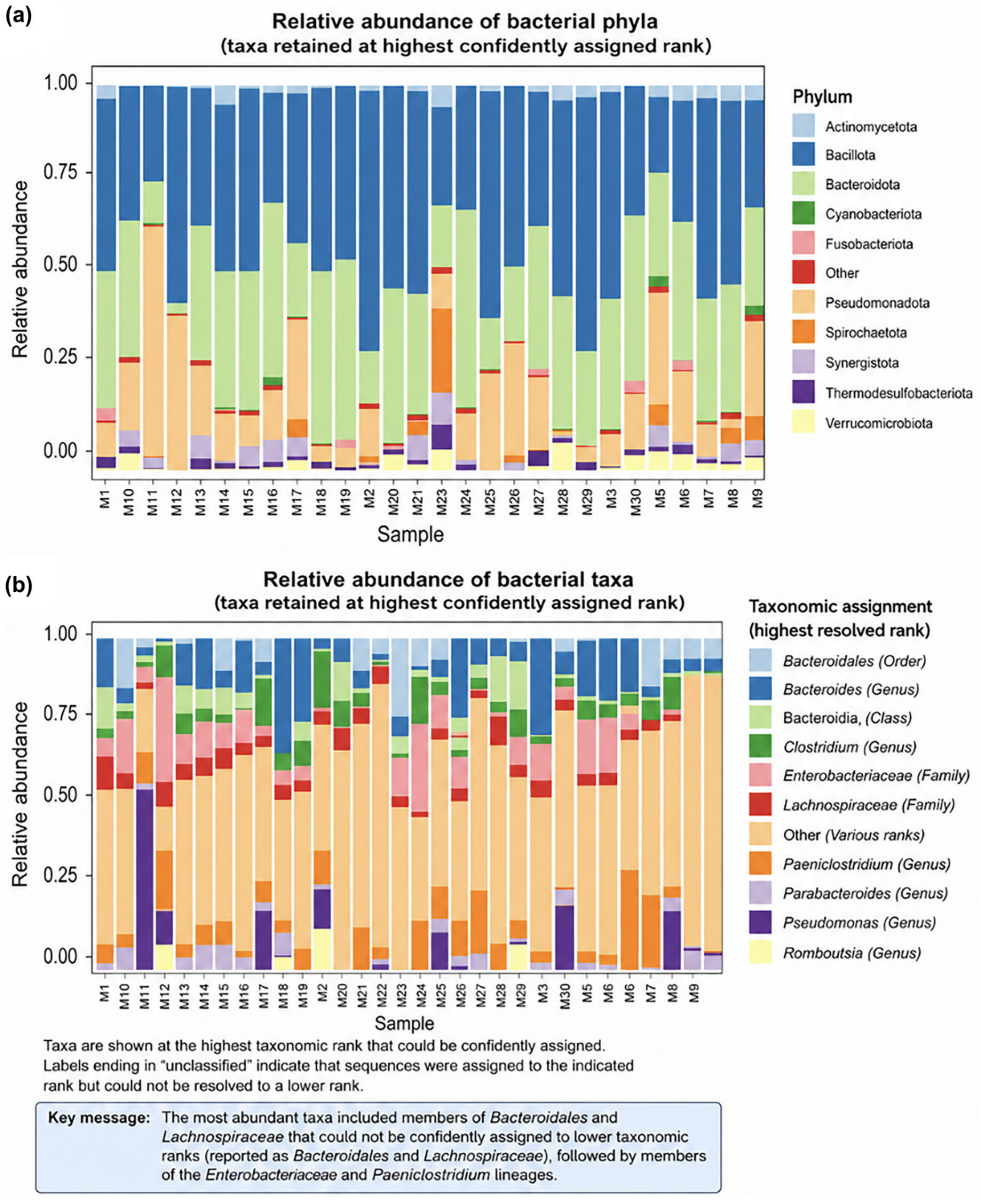
Microbial community by (a) phyla and (b) highest resolved rank composition of the core faecal microbiome of Australian captive pythons.

The microbiome revealed that faecal communities were dominated by members of the phyla Bacillota, formerly known as Firmicutes (∼50%–70%), Bacteroidota also known as Bacteroidetes (∼ 30%–50%) and Pseudomonadota also known as Proteobacteria (∼2%–60%) as shown in Fig. 2. This is consistent with microbial profiles reported in other reptilian gastrointestinal systems (Costello et al. 2010, Xiang et al. 2025). Variation in microbial diversity was observed across pythons, with differences associated with diet and sampling location, suggesting that environmental and husbandry conditions may influence gut microbial diversity. Bacillota was the most abundant phylum, representing over half of the microbial population in many samples, followed by Bacteroidota, which showed variable but substantial representation across samples. Other commonly detected taxa included Actinomycetota which appeared at moderate levels, while Verrucomicrobiota, Synergistota, Thermodesulfobacteriota, and Spirochaetota were present in low relative abundances. Cyanobacteriota and Fusobacteriota were occasionally detected but typically represented minor components of the community. The faecal microbiota was dominated by Gram-positive and Gram-negative fermentative taxa typical of reptilian hosts, with notable individual variation in microbial composition among samples. Across all samples, the gut microbiome was largely dominated by taxa within the *Bacteroidales* and *Clostridium* spp. reflecting a bacterial community characteristic of anaerobic fermentative metabolism. *Bacteroidales* and *Lachnospiraceae*, which were unclassified were the most abundant taxa, followed by members of the *Enterobacteriaceae* and *Paeniclostridium* lineages. In contrast, *Pseudomonas, Romboutsia*, and several unclassified or minor taxa appeared at very low relative abundance, suggesting they play a more transient or niche-specific roles within the gut ecosystem. Sample M1 from the *Morelia spilota cheynei* (jungle carpet python) in Maldon was the only sample reported to lack *Pseudomonas* spp.

Higher relative abundance of *Enterobacteriaceae* within which *Salmonella* belongs to, corresponded with *Salmonella*-positive samples, indicating possible microbial associations. However, there were no statistically significant associations reported between the relative abundance of *Enterobacteriaceae* and *Salmonella* (*β* = 0.875, *p* = 0.351, *q* = 0.996) in MaAsLin2. A limitation of MaAsLin2 are some models are not feasible in predicting weak effects within microbial communities specifically within a small sample size (Mallick et al. 2021). This pattern can be interpreted as a descriptive trend rather than evidence of a significant microbial association. Taxa belonging to the family *Enterobacteriaceae* were detected at low relative abundances in several faecal samples. This suggests that *Salmonella* or closely related genera may have been present but not resolved at the genus level due to the limited discriminatory power of 16S rRNA sequencing. A limitation of this study includes with the advancement of ASVs, which uses DADA2, it has higher specificity and resolution as compared to OTUs generated by MOTHUR in this study (Lehr et al. 2025). The detection of *Enterobacteriaceae* taxa in the microbiome profiles supports the idea that *Salmonella* may remain part of the commensal or transient bacterial community, even when not detected through culture-based isolation. This highlights the sensitivity of 16S rRNA gene sequencing for identifying low-abundance or dormant *Salmonella* populations that might be overlooked by conventional culture methods. In addition, taxonomic units can be generated from both living and dead cells using 16S rRNA sequencing (Kang et al. 2020).

**Figure 3:**
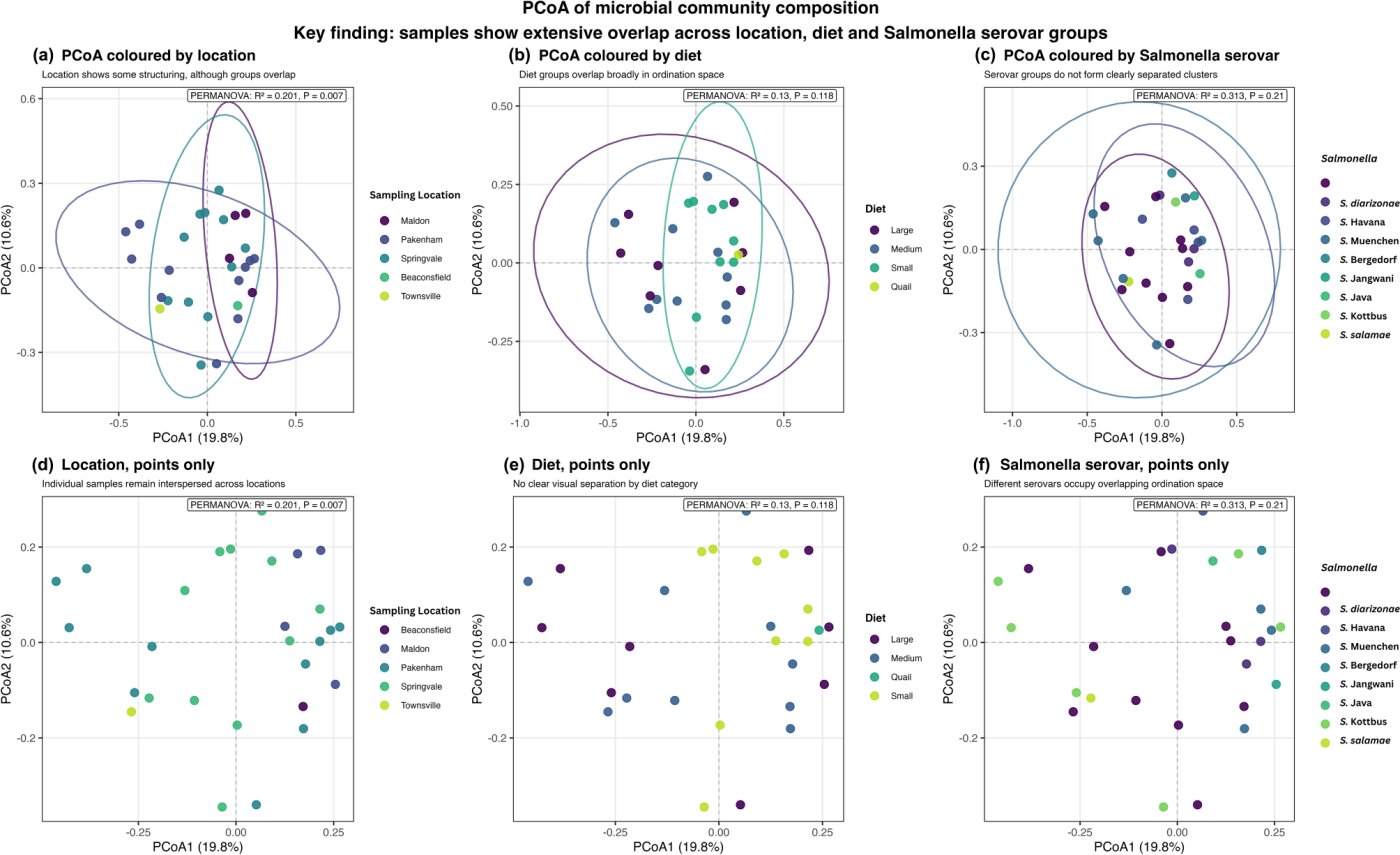
PCoA of 16S rRNA gene sequencing data illustrating microbial community variation in phyla across different factors. PCoA was performed using beta-diversity distances derived from bacterial community profiles. Each plot displays the first two principal components (PCoA1 and PCoA2), which together explain ∼30% of the total variance in the dataset (PCoA1 = 19.8%, PCoA2 = 10.6%). The analysis was performed using relative abundance data at the phylum level. Top row: PCoA plots with 95% confidence ellipses show clustering of microbial communities based on (a) sampling location, (b) diet, and (c) *Salmonella* serovar/subspecies detected in corresponding strains. Distinct groupings and overlap between clusters suggest partial differentiation in microbial community composition among these factors. Bottom row: Corresponding PCA scatterplots without confidence ellipses display the individual sample distribution by (d) sampling location, (e) diet, and (f) *Salmonella* serovar/subspecies. PERMANOVA values are displayed within each panel. Overall, microbial communities exhibited substantial overlap across sampling locations, diet categories, and *Salmonella* serovars, indicating limited separation of community composition based on these factors. Colours indicate categorical groupings as shown in the legends.

The correlation of the degree of sampling location, diet, and *Salmonella* serovars or subspecies with the microbial composition was determined using principal coordinates. Principal Coordinates Analysis (PCoA) plots coloured by sampling location (Panel a) and diet (Panel b) in Fig. 3 showed partial overlap among groups, suggesting some shared microbial profiles across sites and feeding regimes, though samples from Springvale and Pakenham formed tighter clusters, indicating locally consistent microbiota. When distinguished by *Salmonella* serovar or subspecies (Panel c), *S*. Muenchen and *S*. Havana visually tended to group more closely together, reflecting compositional similarity, whereas *S*. diarizonae appeared more distinct, consistent with phylogenetic differentiation observed in genomic analyses. However, PERMANOVA did not detect significant differences (*P* ≥ 0.05) in microbial community composition among *Salmonella* serovars/subspecies. Therefore, these patterns should be interpreted merely as visual trends. Several serovars/subspecies were represented by only a single sample, which limited the statistical analysis to determine differences among groups. PCoA of the faecal microbiomes at the phylum level revealed distinct clustering patterns when coloured by sampling location, diet, and *Salmonella* serovar (top panels). The first two principal components (PCoA1 and PCoA2) explained approximately 30% of the total variance in microbial community composition, with PCoA1 (19.8%) contributing to the separation among samples.

**Figure 4:**
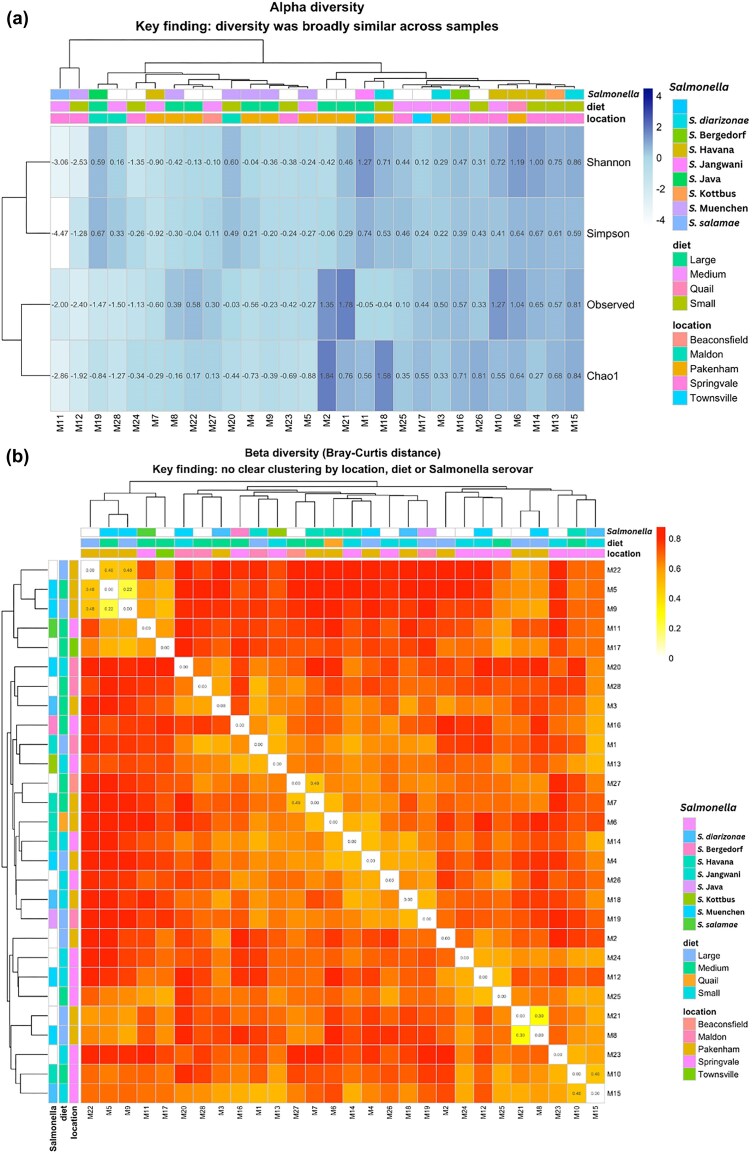
Heatmap showing (a) alpha diversity indices and (b) beta diversity across microbial communities isolated from python samples. For the alpha diversity indices, diversity was assessed using the Shannon, Simpson, observed species richness, and Chao1 indices. Values were standardized (*z*-score transformed) prior to visualization. Colours and numbers represent deviations from the mean diversity value for each metric rather than raw diversity estimates. Positive values indicate samples with higher-than-average diversity, whereas negative values indicate lower-than-average diversity. Clustering was performed using hierarchical methods to group samples based on similarity in diversity profiles. For the beta diversity indices, each cell represents the degree of similarity between two samples. Each cell represents the pairwise dissimilarity between two microbial communities, ranging from 0 (identical communities) to 1 (completely dissimilar communities). Hierarchical clustering was used to group samples according to similarities in community composition. Annotation bars indicate *Salmonella* serovar/subspecies, diet and sampling location.

Alpha diversity determines the complexity and variety of microorganisms within a specific area, habitat, or sample. Alpha diversity indices, including Shannon, Simpson, observed richness, and Chao1, demonstrated inter-individual variability (Shannon and Simpson, *P* = 0.157; Observed richness, *P* = 1.0 Chao1, *P* = 0.480) in microbial richness and evenness among individuals, with certain pythons exhibiting more diverse and complex microbial communities than others as shown in Fig. 4(a). In this study, Alpha diversity was low due to the limited number of sampling locations from where the samples were obtained. Although alpha diversity indices were not statistically different among groups, a trend in colour and numerical differences was observed. These differences suggest that host-specific factors such as species, diet, or sampling location may influence microbial diversity patterns. Both M11 and M12 obtained from a *Morelia spilota cheynei* (jungle carpet python) and *A. maculosa* (spotted python), respectively, in Springvale, Victoria were fed small rats and were reported to exhibit a less diverse microbiome compared to the remaining samples investigated in the study. This analysis highlights differences in microbial richness and evenness among individual python hosts, reflecting potential influences of host species, environment, or health status on microbial diversity.

Beta diversity analysis using Bray–Curtis dissimilarity further illustrated compositional differences in microbial communities across samples in Fig. 4(B). Beta diversity which was tested using PERMANOVA showed significant differences in microbial communities (*P* ≤ 0.006, pseudo-*F* = 1.449, *R*^2^ = 0.201). Beta diversity revealed that the pythons have a diverse set of microbial communities although it came from the same residence. Pairwise PERMANOVA analysis showed that the microbial communities of pythons from Maldon and Springvale differed significantly (adjusted *P* = 0.018), whereas no other pairwise sampling location comparisons remained significant after correction for multiple testing. These findings suggest that geographical sampling locations may influence gut microbial community structure, although the effect was not uniform across all sampled locations. In addition, to determine whether individual sampling locations differed from the remaining sampled locations, sampling location-versus-all-other PERMANOVA comparisons were performed using Bray–Curtis dissimilarity. Maldon significantly differed from all other sampling locations combined (*F* = 1.965, *R*^2^ = 0.070, adjusted *P* = 0.030), while Springvale also significantly differed from all other sampling locations (*F* = 2.001, *R*^2^ = 0.071, adjusted *P* = 0.030). In contrast, Townsville (adjusted *P* = 0.591), Pakenham (adjusted *P* = 0.177), and Beaconsfield (adjusted *P* = 0.759) did not differ significantly from the remaining sampling locations. Overall, the heatmap revealed clusters of pythons sharing similar microbial communities, alongside individuals with distinctly different profiles, indicating a degree of ecological differentiation within the captive population. Multivariate dispersion differed significantly among sampling locations (PERMDISP: *F* = 31.042, *P* = 0.001), indicating that variability within sampling location contributed to the observed differences. Pairwise comparisons showed that Beaconsfield and Townsville exhibited significantly lower dispersion than Maldon, Pakenham, and Springvale (*P* ≤ 0.001), whereas no significant differences (*P* > 0.05) in dispersion were observed among Maldon, Pakenham, and Springvale. These findings suggest that both differences in community composition and differences in community heterogeneity contributed to the observed beta diversity patterns.

### Biofilm cell numbers and specific biofilm formation

The results of the *Salmonella* numbers on polystyrene coupons at 25 °C and 37 °C using full strength and half strength TSB are shown in Table 1. Biofilm cell numbers across all strains were ∼ 5.59–7.30 log CFU cm^−2^ in full-strength TSB and ∼4.82–7.47 log CFU cm^−2^ in half-strength TSB at both temperatures. In full-strength and half-strength TSB, most strains exhibited similar biofilm cell numbers at 25 °C and 37 °C, with no significant differences (*P* ≥ 0.05). *S*. Java S2A and *S*. Typhimurium ATCC 14028 were the only strains that exhibited significantly (*P* ≤ 0.05) higher biofilm cell numbers at 37 °C compared to 25 °C in both full-strength and half-strength TSB. On the contrary, *S*. Muenchen S12A was the only strain that exhibited significantly (*P* ≤ 0.05) lower biofilm cell numbers at 37 °C compared to 25 °C in full-strength TSB. In half-strength TSB, *S*. Havana S7A and *S. diarizonae* S3A were the only strains that exhibited significantly (*P* ≤ 0.05) lower biofilm cell numbers at 37 °C compared to 25 °C in full-strength TSB.

**Table 1:** Biofilm cell numbers and Specific Biofilm Formation of *Salmonella*.

Strain	*Salmonella* numbers (Log CFU/cm^2^)				Specific Biofilm Formation			
	Full-strength TSB		Half-strength TSB		Full-strength TSB		Half-strength TSB	
	25 °C	37 °C	25 °C	37 °C	25 °C	37 °C	25 °C	37 °C
*S*. Jangwani S1A	6.28^BCa^ ± 0.21	6.00^Aa^ ± 0.34	6.28^Ba^±0.20	5.89^ABa^ ± 0.17	0.28^Aa^ ± 0.20	0.05^Aa^ ± 0.37	-0.05^Aa^ ± 0.18	0.69^ABd^ ± 0.36
*S*. Java S2A	5.59^Aa^ ± 0.26	6.36^ABb^ ± 0.04	4.82^Aa^ ± 0.17	6.54^BCDb^ ± 0.23	0.25^Aa^ ± 0.48	0.11^Aa^ ± 0.46	0.82^Aa^ ± 1.49	1.46^Aa^ ± 1.03
*S*. Muenchen S9A	6.37^BCa^ ± 0.37	6.27^ABa^ ± 0.38	6.21^Ba^ ± 0.24	6.06^ABa^± 0.30	0.03^Aa^ ± 0.01	0.09^Aa^ ± 0.03	0.13^Aa^ ± 0.05	0.27^ABb^ ± 0.02
*S*. Muenchen S12A	6.72^Ca^ ± 0.08	5.97^Ab^ ± 0.48	6.25^Ba^ ± 0.22	5.86^ABa^ ± 0.42	0.17^Aa^ ± 0.08	0.19^Aa^ ± 0.02	0.47^Aa^ ± 0.24	0.59^ABa^ ± 0.26
*S*. Havana S7A	6.55^BCa^ ± 0.11	6.14^ABb^ ± 0.48	6.67^BCa^ ± 0.18	6.01^ABb^ ± 0.29	0.20^Aa^ ± 0.04	0.21^Aa^ ± 0.04	0.34^Aa^ ± 0.04	0.29^ABa^ ± 0.09
*S*. Havana S14A	6.83^Ca^ ± 0.24	6.79^ABb^ ± 0.15	6.58^BCa^ ± 0.41	6.67^BCDa^ ± 0.16	0.09^Aa^ ± 0.04	0.17^Aa^ ± 0.07	0.42^Aa^ ± 0.04	0.35^ABb^ ± 0.13
*S*. Umbadah S20A	6.87^Ca^ ± 0.15	6.92^BCa^ ± 0.12	6.82^BCa^ ± 0.21	6.12^ABa^ ± 0.52	0.21^Aa^ ± 0.10	0.21^Aa^ ± 0.09	0.29^Aa^ ± 0.10	0.47^ABa^ ± 0.06
*S. diarizonae* S3A	6.72^Ca^ ± 0.17	6.10^Ab^ ± 0.33	6.52^BCa^ ± 0.12	5.17^Ab^ ± 0.41	0.10^Aa^ ± 0.03	0.04^Aa^ ± 0.13	0.06^Aa^ ± 0.03	0.13^ABa^ ± 0.17
*S. diarizonae* S18A	6.34^BCa^ ± 0.32	6.61^ABCa^ ±0.34	6.37^BCa^ ± 0.20	6.62^BCDa^ ± 0.22	0.16^Aa^ ± 0.02	0.27^Aa^ ± 0.11	0.54^Aa^ ± 0.07	0.73^ABa^ ± 0.22
*S. salamae* S11A	6.72^Ca^ ± 0.26	6.68^ABCa^ ±0.12	6.73^BCa^ ± 0.12	6.11^ABa^ ± 0.53	0.19^Aa^ ± 0.02	0.30^Aa^ ± 0.10	0.42^Aa^ ± 0.11	0.57^ABa^ ± 0.28
*S*. Typhimurium ATCC 13311	6.92^Ca^ ± 0.28	6.71^ABCa^ ±0.25	6.97^Ca^ ± 0.12	7.11^CDa^ ± 0.32	0.19^Aa^ ± 0.02	0.20^Aa^ ± 0.02	0.62^Aa^ ± 0.09	0.70^ABa^ ± 0.30
*S*. Typhimurium ATCC 14028	6.87^Ca^ ± 0.02	7.30^Cb^ ± 0.16	6.80^BCa^ ± 0.02	7.47^Db^ ± 0.07	2.17^Ba^ ± 0.06	3.08^Cb^± 0.43	3.57^Ba^ ± 0.60	5.54^Ca^ ± 1.25

Uppercase letters indicate significant differences (*P* ≤ 0.05) between strains within a specific temperature for biofilm cell numbers or specific biofilm formation, while lowercase letters indicate significant differences (*P* ≤ 0.05) between temperatures within a strain for biofilm cell numbers or specific biofilm formation.

Specific biofilm formation was used to determine *Salmonella* growth and biofilm formation at both temperatures investigated in this study as shown in Table 1. It was evident that biofilm formation was significantly lower by all the reptile-associated strains in comparison to the control, *S*. Typhimurium ATCC 14028 when examined with both full and half-strength TSB. *S*. Typhimurium ATCC 14028 is a better biofilm former in comparison to the other control strain, *S*. Typhimurium ATCC 13311 investigated in this study. This is in agreement with previous studies (Richter et al. 2023). In half-strength TSB, there were significant differences observed between 25 °C and 37 °C in *S*. Jangwani S1A, *S*. Muenchen S9A and *S*. Havana S14A.

## Discussion

Previous studies have reported the presence of *Salmonella* across reptile species but to our knowledge, there aren’t any studies on the relationship between the microbiome of captive python faeces and the presence/absence of *Salmonella*. Our study shows that the microbiome of the captive python faeces differed when compared with the carriage of *Salmonella*, diet, and sampling location. The characterization of 23 *Salmonella* strains of multiple subspecies highlights the genetic heterogeneity of *Salmonella* in captive pythons (Muslin et al. 2025). Studies have reported that *Salmonella* carriage varied across reptiles with the highest observed in snakes (63%) followed by lizards (33.6%), turtles (11.6%), and crocodiles (10.5%) (Muslin et al. 2025). In this study, 68% of faecal samples were reported to carry *Salmonella*, which is comparable to the current literature. However, various factors such as captivity, diet, sampling location and snake species may contribute to the presence /absence of *Salmonella*. Variation of subspecies and serovars in captive reptiles could be attributed to the housing environment (Muslin et al. 2025). The predominance of subspecies *enterica* reflects the common host association of this lineage with warm-blooded vertebrates, yet its persistence in reptiles suggests ecological versatility and adaptation to ectothermic hosts (Bäumler et al. 1998). *S*. Muenchen and *S*. Havana exhibited high ANI values (>99%) within serovars, consistent with previous studies that have shown limited intra-serovar genomic divergence (Kingsley and Bäumler 2000, Gupta et al. 2019). Both these serovars were the most frequently isolated serovars across multiple python species, including *Aspidites melanocephalus* (black-headed python)*, Antaresia childreni* (children’s python), *Morelia spilota* (Murray Darling python), *Liasis olivaceus* (olive python), *Morelia oenpelliensis* (Oenpelli python), and *Antaresia maculosa* (spotted python). This suggests a broad host range and environmental adaptability. *S*. Muenchen strains formed two closely related genomic subclusters, with ANI values of 99.99%–100.00% within subclusters and 99.19%–99.20% between subclusters, indicating modest genomic differentiation within the serovar. Despite these subclusters, all *S*. Muenchen strains remained highly similar genomically, suggesting the presence of at least two closely related lineages rather than substantial divergence. Similarly, *S*. Havana and *S*. Java strains demonstrated minimal genomic variation. Strict host specificity of *Salmonella* serovars were also not evident to individual python species. *S*. Jangwani isolated from *Morelia spilota cheynei* (jungle carpet python) in our study, has been isolated from geckos previously in Malawi and Poland but have not been previously reported in snakes (Zając et al. 2021, Wilson et al. 2024 ). However, this serovar is associated with reptiles. The clustering of subspecies *diarizonae* into a distinct lineage, separated from subspecies *enterica* strains in both phylogenetic and principal component analyses, supports the view that this subspecies represents a reptile-adapted clade with long-term evolutionary separation (Giner-Lamia et al. 2019, Lamas et al. 2021).

The genomic similarity among *S. diarizonae* (S3A, S7B, S15A and S18A) based on the inStrain SNP and ANI phylogenetic tree suggests recent shared ancestry or may reflect transmission to a common source. This pattern aligns with previous evidence of cross-host *Salmonella* transmission among reptiles exposed to common feeding and handling practices (Corrente et al. 2017, Whiley et al. 2017). None of these pythons were housed together refers to pythons not being kept in a same enclosure. However, there were occasions when a pet owner owned more than one python. In these cases, python owners may use the same utensils to feed the pythons possibly resulting in the transmission of *Salmonella* within captive environments. Subspecies *diarizonae* was only isolated from *Aspidites melanocephalus* (black-headed python)*, Antaresia maculosa* (spotted python)*, Morelia oenpelliensis* (Oenpelli python), and *Morelia spilota variegata* (Albino Darwin python), suggesting a possible preference or adaptation towards these reptile hosts. Previous studies have reported subspecies *diarizonae* is a reptile-adapted lineage with enhanced persistence in ectothermic hosts (Giner-Lamia et al. 2019, Lamas et al. 2021). Virulence and environmental adaptability genes were largely conserved across all *Salmonella* isolates, indicating that stress-response mechanisms are not restricted to a single subspecies or serovar.

Distinct microbial compositional differences were observed across sampling locations and dietary regimes. Sampling locations will be expected to influence findings related to the composition of microbial communities due to differences in temperature and humidity. Variation in temperature and humidity will contribute to the differences in environmental microorganisms that the captive pythons are exposed to when they are brought out of their enclosures for a clean or feed. In addition, husbandry differences across pet owners from a sampling location may differ from another pet owner in another sampling location. Pet owners from different sampling locations may be giving a similar diet but the feed might be obtained from another manufacturer, which may also contribute to differences in microbial communities. Pythons fed larger rodents exhibited increased proportions of Bacillota, whereas those fed smaller prey or quail had a higher relative abundance of Pseudomonadota. Quails have been reported to carry Bacillota followed by Bacteroidota and a low abundance of Pseudomonadota (Gomes et al. 2025). The ceca of birds carry >90% of Gram-positive bacteria (Gong et al. 2002). On the contrary, studies have also reported an increase in abundance of Pseudomonadota, while a lower abundance of Bacillota in quails treated with insecticide treatment (Crisol-Martínez et al. 2016). Female quails treated with insecticide were also reported to carry a higher abundance of *Salmonella* compared to male quails (Crisol-Martínez et al. 2016). In this study, the gender of feeder rodents and birds was not recorded. This study only had quail as the diet for an *Aspidites melanocephalus* (black-headed python), which carried *S*. Muenchen. Most captive python owners tend to feed feeder rodents rather than feeder quails limiting the breadth on the carriage of *Salmonella* in captive pythons fed quails. This dietary influence on microbial structure mirrors trends in other vertebrates, where high-protein diets favour Proteobacterial enrichment (Song et al. 2025, Zhu et al. 2025). Pythons carrying *Salmonella* exhibited a slightly reduced microbial evenness, as reflected in lower Shannon and Simpson indices as shown in the alpha diversity values. This suggests that *Salmonella* colonisation may alter the microbial community equilibrium, potentially through competitive exclusion or modulation of gut conditions such as oxygen tension (Sorbara and Pamer 2019, Singh and Koo 2024). Samples with distinct microbial compositions such as M11, M12, and M19 corresponded to hosts infected with genomically divergent *Salmonella* strains, further implying a link between subspecies /serovar and host microbiota diversity. The correlation observed between *Salmonella* detection and the relative abundance of *Enterobacteriaceae* supports the hypothesis that members of this family, which includes *Salmonella, Escherichia*, and *Klebsiella*, share ecological niches within the reptile gut (Janda and Abbott 2021). Samples that were positive for *Salmonella* consistently displayed higher *Enterobacteriaceae* abundance, indicating that *Salmonella* may co-exist with other facultative anaerobes capable of thriving in nutrient-variable environments. This trend aligns with studies showing that *Enterobacteriaceae* enrichment often accompanies pathogen carriage in reptiles (McWhorter et al. 2021, Pees et al. 2023). Although M8 and M20 obtained from *Aspidites melanocephalus* (black-headed Python) lacked *Enterobacteriaciae* from the microbiome analysis, *S*. Muenchen was detected in these samples. The microbiome data may not have been able to detect the *Enterobacteriaciae* family due to low levels, while *S*. Muenchen was detected as it was enriched using culture-based techniques. Previous studies have suggested that rodent-based diets in reptiles and captive-bred species are associated with increased shedding of *Salmonella*. Approximately 1/3 of pythons fed a rodent-based diet in this study lacked *Salmonella*. A potential limitation of this study is that reptiles have been associated with intermittent shedding of *Salmonella*, which may result in some of the samples lacking *Salmonella* (Goupil et al. 2012, McWhorter et al. 2021). This is due to the sampling being conducted once only from the python of concern.

Both Alpha and Beta diversity analyses demonstrated clear geographic clustering of python microbiomes, particularly among pythons from Pakenham and Springvale. This spatial structuring may reflect differences in husbandry practices, enclosure conditions, or exposure to local microbial reservoirs. Beta diversity, assessed via Bray–Curtis dissimilarity, revealed that pythons from Maldon and Townsville hosted more distinct microbial assemblages, consistent with environmental influences on microbiome composition. This is evident with the significant differences (*P* ≤ 0.006) in microbial communities among geographical sampling locations. This suggests that sampling locations may influence gut microbial community composition. The differences of certain python microbiomes, such as those from M22 and M25 from Pakenham and Springvale, Victoria, respectively, further highlight the role of environmental isolation in shaping gut microbial communities (Colston et al. 2025). PCoA results supported the diversity metrics by illustrating distinct groupings among samples from different sites and diets, suggesting that environmental and management factors influence microbial composition. Alpha and beta diversity analysis added an ecological dimension to these findings, quantifying within and between-sample variation. Alpha diversity showed that microbial richness and evenness were relatively consistent across sites, while beta diversity highlighted differences in community composition between populations, particularly for pythons from Maldon and Townsville. Together, these complementary analyses confirmed that while the python gut microbiome is dominated by similar bacterial phyla, the relative structure and diversity of those communities differ according to environmental context, husbandry conditions, and host-associated factors.

Persistence of *Salmonella* in reptile enclosures can be source of cross-contamination while handling reptiles. Polystyrene is commonly used to create customized enclosure features such as hides and backgrounds due to its ease of carving and shaping. Many reptile enthusiasts use it to construct rock walls and hedges. Thus, our study also sought to determine the biofilm-forming abilities of some of these reptile-associated strains on polystyrene at two temperatures relevant to reptile enclosures under nutrient-limiting conditions. Some studies have reported that 25 °C tends to be a better temperature than 37 °C for food and human-associated *Salmonella* biofilm formation (Yin et al. 2018, Akinola et al. 2020, Lv et al. 2026). Reptile-associated *Salmonella* strains were consistently weaker biofilm formers than the reference strains. Reptiles, as ectotherms, might not experience the same selective pressures on biofilm formation as warm-blooded hosts. This may be attributed to host-associated ecological factors. These ecological differences may influence adhesion capabilities, environmental persistence strategies, and biofilm gene expression. Previous studies have also reported that the reference strain *S*. Typhimurium ATCC 13311 is a better biofilm former than reptile-associated *Salmonella* strains (Sarjit et al. 2023). Among all strains, *S*. Typhimurium ATCC 14028 produced the highest biofilm biomass observed under both full-strength and nutrient-limited conditions. Its consistently strong performance across both the culture-based and crystal violet assays highlights its utility as a reliable biofilm-forming control strain. These findings are consistent with previous literature identifying ATCC 14028 as a model strain capable of forming dense biofilms under various environmental pressures (Steenackers et al. 2012). The enhanced biofilm formation observed under low-nutrient conditions, particularly at 37 °C across *S*. Java S2A and *S. diarizonae* S18A, was reflected in both the specific biofilm formation (SBF) values and optical density-based biomass quantification. This provides evidence that nutrient stress and elevated temperature act as environmental cues for surface adherence and biofilm maturation, potentially involving known regulatory pathways such as quorum sensing or cyclic-di-GMP signalling (Zogaj et al. 2001). A recent study conducted in Italy on monospecies biofilm formation of reptile-associated *Salmonella* from snakes (royal snake, bull snake, gerrohsaurus major, false coral snake, coral snake and royal python) at 37 °C reported ∼9–10 log CFU units after 48 h of incubation (Galgano et al. 2023). The longer duration of exposure and strain variation possibly attributed to different hosts and geographical sampling location may have contributed to higher biofilm cell numbers. Subspecies *enterica-associated* strains were generally poorer biofilm formers than strains belonging to subspecies *salamae* and *diarizonae*. In a previous study, subspecies *enterica* tended to carry a higher number of biofilm-associated genes compared to subspecies *arizonae* and *salamae*, which might be negatively correlated with biofilm formation on polystyrene (Sarjit et al. 2023).

The relatively small sample size within the two states and focus on captive python populations limit generalisation to other snake species. We acknowledge that details of management practises of pythons in reptile enclosures and the age of reptiles are unknown in this study. Future studies should focus on a broader range of reptile hosts, such as lizards, geckos, and turtles, which are known reservoirs of *Salmonella* and possess distinct microbial community structures. Further details on management practices, age of reptiles, and frequency of defecation of reptiles may correlate with the microbiota and carriage of *Salmonella*. Understanding of the ecology of *Salmonella* colonization in captive pythons could be enhanced by investigating the microbiome of the reptile gut.

Subspecies *enterica* with serovars of *S*. Bergedorf, *S*. Havana, *S*. Jangwani, *S*. Java, *S*. Kottbus, *S*. Muenchen, and *S*. Umbadah, as well as subspecies *S. diarizonae* and *S*. salamae were identified from captive pythons. The diversity of isolates across host species and geographic sampling locations highlights the widespread distribution and genetic variability of *Salmonella* within the captive python population. The faecal microbiome is dominated by Bacillota, Bacteroidota, and Pseudomonadota, with variation observed across sampling location, diet, and associated *Salmonella* serovars/subspecies. All reptile-isolated strains were weak biofilm formers. These findings will be useful for public health strategies, particularly in agricultural, veterinary, and domestic settings where reptile-human interactions occur. In addition, it emphasises the importance of monitoring gut community composition to understand how commensal and pathogenic bacteria interact within captive reptiles. Extending this approach to other reptilian hosts could further elucidate host-specific microbiome-pathogen dynamics and inform targeted management strategies for reducing zoonotic risk in reptile husbandry.

## Supplementary Material

fiag084_Supplemental_File
