# Supplementary material for: The faecal microbiome and carriage of Salmonella in Australian captive pythons
Source: FEMS Microbiol Ecol. 2026 Jul 29;102(8):fiag084. doi: 10.1093/femsec/fiag084 (PMC13441174; doi:10.1093/femsec/fiag084)
Supplement: Supplementary file 1 [file fiag084_supplemental_file.docx]

**Table S1.** Environmental Roles of Virulence-Associated Genes Identified in *Salmonella* from Captive Australian Pythons.

| Gene | Function | Environmental or Adaptive Role | Reference |
| --- | --- | --- | --- |
| *grpE* | Co-chaperone in the DnaK–DnaJ–GrpE complex | Assists in protein folding and repair under heat shock and oxidative stress, enhancing bacterial survival during temperature fluctuations and desiccation. | (Li et al., 2024) |
| *dnaK* | Major heat shock protein (Hsp70 family) | Refolds denatured proteins and protects against heat and osmotic stress; critical for maintaining protein stability under fluctuating environmental and host conditions. |  |
| *rpoH* | Sigma factor σ^32 (heat shock regulator) | Activates transcription of heat shock genes (*dnaK*, *grpE*, *groEL*), enabling adaptation to temperature stress and rapid shifts between host and environmental niches. |  |
| *proV* | Component of the ProU transport system | Functions in osmoregulation by importing compatible solutes such as glycine betaine and proline, protecting cells from desiccation and high osmolarity. |  |
| *rpoS* | Sigma factor σ^S (general stress response regulator) | Governs expression of stress-related genes, enhancing survival during nutrient deprivation, oxidative stress, acid stress, and desiccation—key for environmental persistence. | (Nickerson & Curtiss, 1997) |
| *cfa* | Cyclopropane-fatty-acyl-phospholipid synthase | Modifies membrane fatty acids to increase rigidity and acid resistance, supporting survival in acidic environments such as the gastrointestinal tract. | (Chen et al., 2014) |
| *fabA* | β-hydroxydecanoyl-ACP dehydratase/isomerase | Involved in unsaturated fatty acid biosynthesis; maintains membrane fluidity and structural integrity under temperature and nutrient stress. |  |
| *otsA* | Trehalose-6-phosphate synthase | Catalyses the first step in trehalose synthesis; trehalose acts as a protectant during desiccation, osmotic stress, and heat exposure by stabilising proteins and membranes. | (Li et al., 2024) |
| *otsB* | Trehalose-6-phosphate phosphatase | Completes trehalose biosynthesis; contributes to osmoprotection and desiccation tolerance, promoting long-term survival on dry surfaces such as reptile substrates. |  |
| *rpoE* | Sigma factor σ^E (envelope stress regulator) | Maintains outer membrane integrity under environmental stress (heat, oxidative, or antimicrobial); crucial for resistance to envelope damage. | (Miticka et al., 2003) |
| *recA* | Recombinase A protein | Central to DNA repair and homologous recombination; enables recovery from UV radiation, oxidative damage, or desiccation-induced DNA breaks. | (Mayola et al., 2014) |
| *cspA* | Cold shock protein A | RNA chaperone that stabilises transcription and translation during temperature downshifts; supports growth under cold environmental conditions. | (Ricke et al., 2018) |
| *ompC* | Outer membrane porin C | Regulates membrane permeability under varying osmotic conditions; facilitates solute uptake and environmental adaptability. | (Ray et al., 2019) |
| *ompF* | Outer membrane porin F | Works with OmpC to control diffusion of small molecules; expression responds to osmotic stress and antimicrobial exposure, aiding environmental survival. |  |

**Table S2.** Metadata summary of *Salmonella* isolates, including strain identification, host source, collection date, and sampling location.

| **Microbial ID for microbiome** | **Matching *Salmonella* Isolate ID** | **Strain** | **Source** | **Diet** | **Date of Isolation** | **Location** | **Owner ID** |
| --- | --- | --- | --- | --- | --- | --- | --- |
| M1 | S1A | *S*. Jangwani | Jungle Carpet Python  *(Morelia spilota cheynei*) | Large Rat | 14^th^ August 2024 | Maldon, Victoria. | 1 |
| M2 | S2A | *S*. Java | Water Python  (*Liasis fuscus*) | Large Rat | 9^th^ September 2024 | Phillip Island, Victoria. | 7 |
| M3 | S3A | *S. diarizonae* 50k:z | Black-Headed Python (*Aspidites melanocephalus*) | Medium Rat | 15^th^ March 2025 | Pakenham, Victoria. | 6 |
| M4 | S4A | *S*. Muenchen | Murray Darling Python (*Morelia spilota*) | Medium and Large Rat | 15^th^ March 2025 | Pakenham, Victoria. | 2 |
|  | S4B | *S*. Muenchen |  |  |  |  |  |
| M5 | S5A | *S*. Muenchen | Black-Headed Python (*Aspidites melanocephalus*) | Quail | 15^th^ March 2025 | Pakenham, Victoria. | 2 |
| M6 | S6A | *S*. Havana | Oenpelli Python  (*Morelia oenpelliensis*) | Medium Rat | 15^th^ March 2025 | Pakenham, Victoria. | 2 |
| M7 | S7A | *S*. Havana | Oenpelli Python  (*Morelia oenpelliensis*) | Medium Rat | 15^th^ March 2025 | Pakenham, Victoria. | 2 |
|  | S7B | *S. diarizonae* 50k:z |  |  |  |  |  |
| M8 | S8A | *S*. Muenchen | Black-Headed Python (*Aspidites melanocephalus*) | Medium Rat | 15^th^ March 2025 | Pakenham, Victoria. | 2 |
| M9 | S9A | *S*. Muenchen | Black-Headed Python (*Aspidites melanocephalus*) | Medium Rat | 15^th^ March 2025 | Pakenham, Victoria. | 2 |
| M10 | S10A | *S*. Havana | Olive Python  (*Liasis olivaceus*) | Small Rat | 15^th^ March 2025 | Springvale, Victoria. | 3 |
| M11 | S11A | *S. salamae* 21:z10:[z6] | Jungle Carpet Python  (*Morelia spilota cheynei*) | Small Rat | 15^th^ March 2025 | Springvale, Victoria. | 3 |
| M12 | S12A | *S*. Muenchen | Spotted Python  (*Antaresia maculosa*) | Small Rat | 15^th^ March 2025 | Springvale, Victoria. | 3 |
| M13 | S13A | *S*. Kottbus | Stimson’s Python  (*Antaresia stimsoni*) | Small Rat | 15^th^ March 2025 | Springvale, Victoria. | 3 |
| M14 | S14A | *S*. Havana | Children’s Python  (*Antaresia childreni*) | Medium Rat | 15^th^ March 2025 | Springvale, Victoria. | 3 |
| M15 | S15A | *S. diarizonae* 50k:z | Spotted Python  (*Antaresia maculosa*) | Small Rat | 15^th^ March 2025 | Springvale, Victoria. | 3 |
|  | S15B | *S*. Kottbus |  |  |  |  |  |
| M16 | S16A | *S*. Bergedorf | Black-Headed Python (*Aspidites melanocephalus*) | Large Rat | 15^th^ March 2025 | Springvale, Victoria. | 3 |
| M17 | S17A | None | Jungle Carpet Python  (*Morelia spilota cheynei*) | Small Rat | 15^th^ March 2025 | Townsville, Queensland. | 4 |
| M18 | S18A | *S. diarizonae* 38:(k):1,5,7 | Albino Darwin Python (*Morelia spilota variegata*) | Large Rat | 15^th^ March 2025 | Pakenham, Victoria. | 6 |
| M19 | S19A | *S*. Java | Water Python  (*Liasis fuscus*) | Large Rat | 22^nd^ June 2025 | Maldon, Victoria. | 1 |
| M20 | S20A | *S*. Umbadah | Black-Headed Python (*Aspidites melanocephalus*) | Small Rat | 22^nd^ June 2025 | Maldon, Victoria. | 1 |
|  | S20B | *S*. Muenchen |  |  |  |  |  |
| M21 | None | None | Black-Headed Python (*Aspidites melanocephalus*) | Medium Rat | 15^th^ March 2025 | Pakenham, Victoria. | 2 |
| M22 | None | None | Black-Headed Python (*Aspidites melanocephalus*) | Small Rat | 15^th^ March 2025 | Pakenham, Victoria. | 2 |
| M23 | None | None | Children’s Python  (*Antaresia childreni*) | Medium Rat | 15^th^ March 2025 | Springvale, Victoria. | 3 |
| M24 | None | None | Children’s Python  (*Antaresia childreni*) | Medium Rat | 15^th^ March 2025 | Springvale, Victoria. | 3 |
| M25 | None | None | Murray Darling Python (*Morelia spilota*) | Large Rat | 15^th^ March 2025 | Springvale, Victoria. | 3 |
| M26 | None | None | Darwin Python (*Morelia spilota variegata*) | Large Rat | 15th March 2025 | Springvale, Victoria. | 3 |
| M27 | None | None | Woma Python  (*Aspidites ramsayi*) | Medium Rat | 15th March 2025 | Beaconsfield, Victoria. | 5 |
| M28 | None | None | Stimson’s Python  (*Antaresia stimsoni*) | Large Rat | 15th March 2025 | Maldon, Victoria. | 1 |

**Figure S1.** Principal component analysis (PCoA) of *Salmonella* generated from core-genome single nucleotide polymorphisms identified using Snippy. Each point represents an individual strain coloured according to serovar or subspecies.

A

B

**Figure S2.** Phylogeny of *Salmonella* based on (A) ANI and (B) SNP tree
